# Investigation into using resonant frequency measurements to predict the mechanical properties of Ti-6Al-4V manufactured by selective laser melting

**DOI:** 10.1038/s41598-019-45696-w

**Published:** 2019-06-26

**Authors:** Mark A. Todd, James Hunt, Iain Todd

**Affiliations:** 1 Department of Materials Science and Engineering, University of Sheffield, Sir Robert Hadfield Building, Mappin Street, Sheffield, S1 3JD United Kingdom; 2 Nuclear Advanced Manufacturing Research Centre, University of Sheffield, Advanced Manufacturing Park, Brunel Way, Rotherham, S60 5WG United Kingdom; 3 Advanced Manufacturing Research Centre, University of Sheffield, Advanced Manufacturing Park, Wallis Way, Rotherham, S60 5TZ United Kingdom

**Keywords:** Aerospace engineering, Materials science

## Abstract

There is a need to qualify additively manufactured parts that are used in highly regulated industries such as aerospace and nuclear power. This paper investigates the use of resonant ultrasound measurements to predict the mechanical properties of Ti-6Al-4V manufactured by selective laser melting using a Renishaw AM 250. It is first demonstrated why *R*^2^ should not be used to assess the predictive capability of a model, before introducing a method for calculating predicted *R*^2^, which is then used to assess the models. It is found that a linear model with the resonant frequency peaks as predictors cannot be used to predict elongation at failure or reduction in area. However, linear models did demonstrate better predictive capabilities for Young’s modulus, yield strength, and especially ultimate tensile strength.

## Introduction

The use of additive manufacturing is rapidly increasing, with an estimated increase in the value of parts produced of 32.4% in 2017^1^. Highly regulated industries such as aerospace and nuclear need to be able demonstrate that parts manufactured using additive techniques achieve satisfactory performance when compared to parts made by more established processing routes.

Process compensated resonant testing (PCRT)^2^ is a method of non-destructive evaluation (NDE) that measures the acoustic resonance of a part over a certain spectrum, and compares the resonant frequency peaks of the part to a training library of parts in order to classify the measured part using the Mahalanobis-Taguchi System^3^.

Sidambe *et al*.^4^ described a “strong correlation” (*R*^2^ = 0.8384) between one of the resonant frequency peaks and the 0.2% yield stress of Ti-6Al-4V parts manufactured using metal injection moulding (MIM); the resonant frequency peaks were measured using PCRT equipment. Sidambe proposed that a linear model using a single resonant frequency peak as the predictor could be used to make predictions about the 0.2% yield stress. They then used their model to predict the 0.2% yield stress of a test population, and found that the error between predicted and experimental results was less than 10%. It should however be noted that error of 10% appears to be greater than the maximum difference observed between the experimental results in their test population.

The aim of this study was to investigate whether the shift in resonant frequency peaks could be used to make predictions about the mechanical properties of Ti-6Al-4V parts manufactured using selective laser melting (SLM), with a view to using this technique to non-destructively evaluate additively manufactured components.

This article starts with an explanation of the experimental method used for obtaining the tensile test data (response variables) and the resonant frequency data (predictors). Next, a brief introduction to the statistical concepts of leave one out cross-validation (model selection), predicted *R*^2^, and the lasso (variable selection) are presented for the reader who is unfamiliar with those concepts. This is followed by a description of the method used for creating the statistical models. The experimental results are then presented, followed by the results of the statistical models and a discussion of those results. The final part of this work gives the authors’ conclusions about the usefulness of resonant frequency data in predicting tensile properties.

## Method

### Experimental method

#### Selective laser melting

A batch of 60 mechanical test blanks was additively manufactured using a Renishaw AM 250 selective laser melter. The AM 250 uses a 50 ms pulsed ytterbium fibre laser of wave length 1060 nm. As these parts were primarily intended for fatigue testing, the part geometry was based on the 12.5 mm^2^ cross section FCE type A cylindrical fatigue specimen detailed in BS EN 6072:2010^5^. To enable the parts to be machined to final size before mechanical testing, extra material was included by adding an offset of 0.5 mm to all surfaces in the CAD model. The parts were designed around a fatigue geometry to support another element of work under this grant, however tensile tests were used in this part of the work due to significantly narrower distribution of results obtained by tensile testing than by fatigue testing.

**Figure 1:**
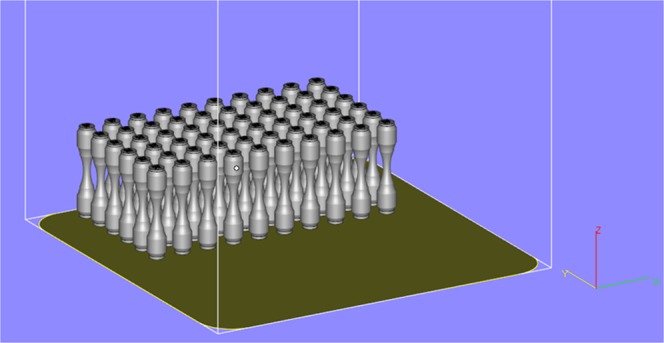
Screen shot from Magics showing specimen layout and Cartesian axes.

Following creation of the 3D CAD representation of the test geometry the file was processed using Materialise Magics. Specimen samples were located on the build plate as indicated in Fig. 1. Individual specimen ID’s were extruded on to the top surface of each specimen. These specimens required no additional support structures, however, the bottom face was extruded by 0.5 mm to facilitate removal via wire electro-discharge machining (EDM) following completion of the build.

The parts were manufactured using a 30 μm layer thickness, and a meander scan strategy with a 67° hatching direction rotation between layers. The laser power, exposure time, point distance, and hatch distance values are given in Table 1. The mean travel speed of the laser spot is inferred by the exposure time, point distance, and inter-point travel speed. The powder used was Renishaw Ti-6Al-4V ELI-0406 powder, which has a nominal particle size range of 15–45 μm. The parts were built with the longitudinal axis parallel to the machine Z-axis.

**Table 1:** Selective laser melting key build parameters.

	Hatching	Boarder scan
Laser power	200 W	100 W
Exposure time	50 μs	40 μs
Point distance	75 μm	45 μm
Inter-point travel speed	5 m/s	5 m/s
Hatch distance	65 μm	60 μm

The parts were removed from the build plate using EDM prior to annealing to enable resonant testing in both the as built and annealed conditions. The annealing heat treatment, which was performed in a vacuum furnace, was:

1. Evacuate the chamber.
2. Heat to 350 °C in 1 hour.
3. Hold at 350 °C for $\frac{1}{2}$ hour.
4. Heat to 850 °C in 1 hour.
5. Hold at 850 °C for 1 hour.
6. Turn-off heating and allow to furnace cool.
7. Turn-off the vacuum pumps once the temperature was below 100 °C.

#### Resonant frequency measurements

**Figure 2:**
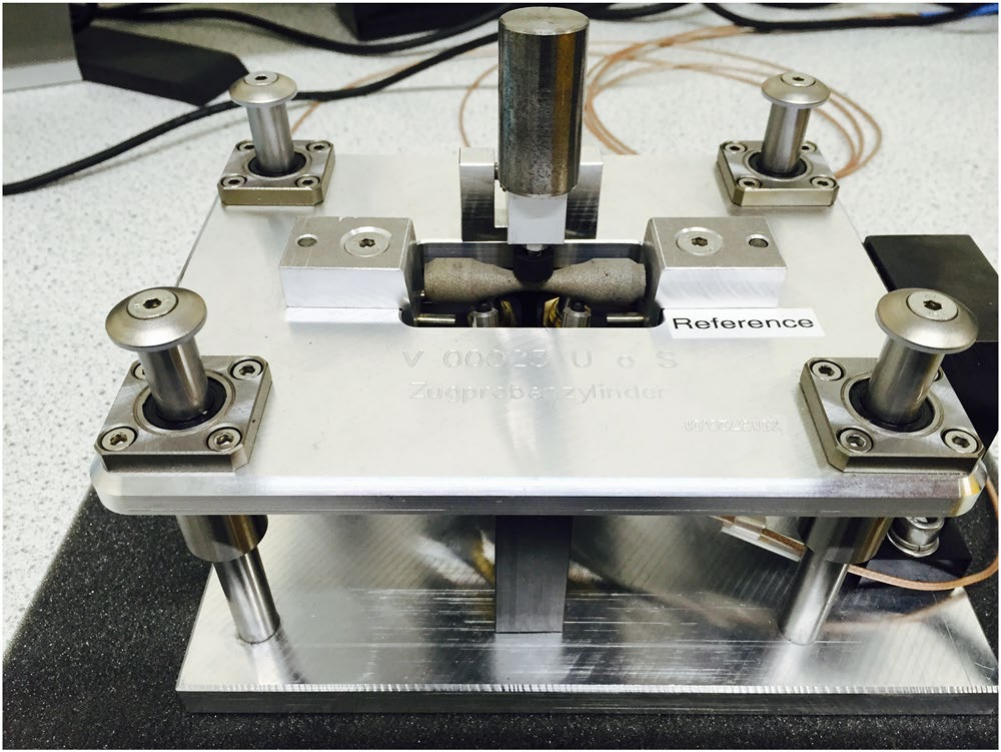
Resonance ultrasound testing jig.

The resonant ultrasound testing was performed using a system provided by Vibrant GmbH (Elz, Germany), which was designed for PCRT. The measurement system included a piezoelectric driver for inducing the resonance and two piezoelectric sensors for measuring the resonance. The driver and sensors were brought into contact with the part by placing the part in a purpose built jig that was provided with the measurement system (see Fig. 2). The primary purpose of the jig was to ensure consistent placement of the driver and sensors.

Resonance ultrasound measurements were taken from the parts in the as built state and after annealing. The frequency scanning ranges were selected by the RUT instrument manufacturer as being from 500 Hz to 226.5 kHz for the as built parts, and from 500 Hz to 600.5 kHz for the annealed parts. The measurement of 8 resonant frequency peaks was selected by the instrument manufacturer for parts in the as built state and 17 for after annealing. Further details about the instrument manufacturer’s process for selecting the resonant frequencies can be found in Sloan *et al*.^2^. The driver and sensors were controlled by the instrument manufacturer’s data acquisition system, which was in turn connected to a personal computer (PC) running the manufacturer’s Quasar/Galaxy software. The Galaxy software analyzed the sensor data to identify the resonant frequencies of the part. When a peak could not be identified during data collection the software issued a warning message, in which case the scan was repeated for that part. This would sometimes enable the identification of all peaks, but would other times still lead to incomplete results, in which case the scan was repeated for a third and final time.

#### Tensile

To assess the mechanical properties of the samples, fifteen parts were sent to Exova Ltd (Middlesborough, UK) for room temperature tensile testing according to ASTM E8^6^. The samples were machined by the testing company prior to performing the tensile testing. The machined sample dimensions are according to specimen 4 of figure 8 in ASTM E8^6^ for test specimens with a gauge length four times the diameter (gauge length = 16 mm, diameter = 4 mm).

### Statistical method

#### Introduction to cross-validation, predicted *R*^2^, and the lasso

An often used statistic to assess the goodness of fit of a statistical model, and to select between different models, is *R*^2^. *R*^2^ is the ratio of variability in the response variable that can be explained by the model to the total variability in the response variable. The total variability in the response variable is measured by the total sum of squares (*TSS*). The variability that can be explained by the model is equal to the TSS less the residual sum of squares (*RSS*) (1), where *y*_*i*_ is the value of the *i*^*th*^ response variable, ${\hat{y}}_{i}$ is the predicted value of the *i*^*th*^ response variable, and $\bar{y}$ is the mean value of the response variables.

1 $${R}^{2}=\frac{TSS-RSS}{TSS}=1-\frac{RSS}{TSS}=1-\frac{{\sum }_{i=1}^{n}\,{({y}_{i}-{\hat{y}}_{i})}^{2}}{{\sum }_{i=1}^{n}\,{({y}_{i}-\bar{y})}^{2}}$$

*R*^2^ is often seen as being relatively easy to interpret, as it always takes a dimensionless value between 0 and 1, with 0 indicating no fit whatsoever, and 1 indicating a model with a perfect fit to the data. However, a significant problem with *R*^2^ is that the measure improves in the presence of over-fitting. Over-fitting occurs when the model complexity is increased in an attempt to reduce the residual error that is due to noise. Unfortunately, by reducing the residual error for the training set, this can increase the error when making predictions. For example, consider an experiment to measure the thermal expansion of a material over some range where the expansion is actually a linear function of temperature (*l* = 8.64 × 10^−6^ ⋅ *T* + 1), but where the length measurement is subject to a Gaussian error. Table 2 shows some randomly generated data for such an experiment.

**Table 2:** Randomly generated data for the measured length of a heated sample with Gaussian measurement noise.

Temperature (°C)	Observed length (m)
0	0.9999374
10	1.0001048
20	1.0000892
30	1.0004187
40	1.0003786
50	1.0003500
60	1.0005671
70	1.0006786
80	1.0007488
90	1.0007471
100	1.0010152

**Figure 3:**
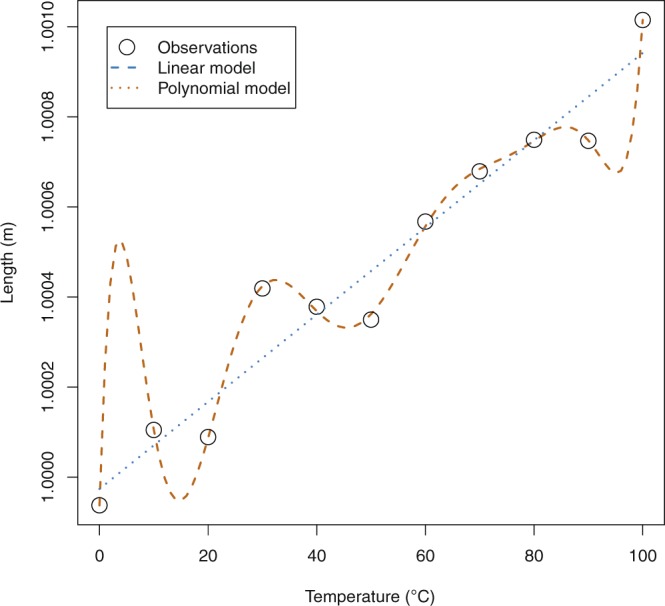
Computer simulated measurements from a linear system with Gaussian noise. The linear model is an accurate model for the system, but is not able completely remove the residual error. The polynomial model is a poor approximation to the underlying regression, but through extreme over-fitting is able to almost eliminate the residual error.

Due to the Gaussian noise in the measurement of the length, the residual error can be reduced by over-fitting. Figure 3 shows the case where both a linear model and a 9th order polynomial have been fitted to the ten data points of Table 2. The linear model (Eq. 2) has an *R*^2^ value of 0.9447, indicating an excellent fit.

2 $$l=9.67757\times {10}^{-6}\cdot T+0.999974$$

However, the polynomial model (Eq. 3) has an *R*^2^ value of 0.9996, indicating an almost perfect fit.

3 $$\begin{array}{rcl}l & = & 1.26661\times {10}^{-17}\cdot {T}^{9}-6.01082\times {10}^{-15}\cdot {T}^{8}\\  &  & +1.20340\times {10}^{-12}\cdot {T}^{7}-1.31849\times {10}^{-10}\cdot {T}^{6}\\  &  & +8.56762\times {10}^{-9}\cdot {T}^{5}-3.33907\times {10}^{-7}\cdot {T}^{4}\\  &  & +7.49217\times {10}^{-6}\cdot {T}^{3}-8.64524\times {10}^{-5}\cdot {T}^{2}\\  &  & +3.92371\times {10}^{-4}\cdot T+0.999937\end{array}$$

Using the *R*^2^ measure, the polynomial model would appear to be better. But it is important to note that whilst the polynomial model is indeed a better fit for this training data, if this model is used to make predictions of sample length based on temperature, then on average the linear model will perform better. For example, the observed value at 30 °C is 1.000419 m. The polynomial model predicts a mean sample length of 1.000424 m at 30 °C. The linear model predicts a mean length of 1.000264 m at the same temperature. The true mean length of the underlying model used in the simulation at 30 °C is actually 1.000259 m. So whilst some repeat measurements at 30 °C may be closer to the value predicted by the polynomial model, on average the measurements at 30 °C will be closer to the value predicted by the linear model.

There are a number of available statistical techniques (see James *et al*.^7^ for some examples) that can be used to assess the ability of a model to make predictions and help the designer to avoid over-fitting; one class of methods is cross-validation. In cross-validation the training data is first split into groups. Then all but one of the groups are used to fit the statistical model. The excluded group is then used as a test set to assess the performance of the fitted model. The excluded group is then returned to the training set, and the process repeated with a different excluded group. This process continues until all groups have been used as the test set once. If the training data is split into groups of one, the method is referred to as leave-one-out cross-validation (LOOCV).

One metric that can be calculated using leave-one-out cross-validation to assess the predictive power of a model is the prediction sum of squares (PRESS)^8^. The PRESS is calculated as the sum of the squared differences between the actual value of the response variable (*y*_*i*_) and the value predicted by the model fitted using the set excluding the value being predicted (${\hat{y}}_{(i)}$) (4). The PRESS can also be extended to the more general case of cross-validation, in which case it is the sum of the squared differences between the actual response variable value and the value predicted by the model fitted using the test set excluding all members of the same group as the member being predicted. If the PRESS statistic is divided by the population size of the training set, this mean cross-validation error becomes an estimator of the test mean squared error (MSE).

4 $$PRESS=\sum _{i=1}^{n}\,{({y}_{i}-{\hat{y}}_{(i)})}^{2}$$

Whilst the estimated test MSE is a widely used performance measure, interpretation can be difficult as the magnitude of the MSE will depend upon the magnitude of the response variable and will have squared units of the response variable. To put the PRESS statistic into perspective, it can be useful to compute the PRESS of the null model. The null model is the model with all of the coefficients set to zero, which is equal to the mean of the response variable. The PRESS of the null model (5) is the sum of the squared differences between the actual value of the response variable (*y*_*i*_) and the mean value of the response variable excluding the samples from the same group as the sample currently being predicted (${\bar{y}}_{(i)}$).

5 $$PRES{S}_{NULL}=\sum _{i=1}^{n}\,{({y}_{i}-{\bar{y}}_{(i)})}^{2}$$

The total sum of squares that appears in Eq. 1 can also be considered as the sum of squares of the null model, which makes *R*^2^ a measure that compares the error (sum of squares) of the model to the error of the null model. Following this definition of *R*^2^, the predicted *R*^2^ can be defined using LOOCV, which replaces the numerator with the PRESS of the model, and the denominator with the PRESS of the null model (6). It should be noted whilst the maximum value of predicted *R*^2^ is one, unlike *R*^2^, predicted *R*^2^ can take negative values. This will be the case if the model has a higher sum of squares error than the null model when tested using LOOCV.

6 $${R}_{PRED}^{2}=1-\frac{PRESS}{PRES{S}_{NULL}}=1-\frac{{\sum }_{i=1}^{n}\,{({y}_{i}-{\hat{y}}_{(i)})}^{2}}{{\sum }_{i=1}^{n}\,{({y}_{i}-{\bar{y}}_{(i)})}^{2}}$$

If the predicted *R*^2^ is calculated for the example models in Fig. 3, the value for the linear model is 0.93, suggesting that the model is a good approximation for the underlying regression and can be used with some confidence to make predictions about sample length based on temperature. However, the predicted *R*^2^ value for the polynomial model is −119, which indicates that the polynomial model is a poor approximation to the underlying system, and should not be used to make predictions about sample length based on temperature measurements.

In the case of a polynomial model with over-fitting, the over-fitting can be reduced by reducing the order of the polynomial without affecting the number of predictors used in the model. In the case of a multiple linear regression, LOOCV still enables the assessment of model over-fitting, but this then leads to the question of how select the predictors to remove from the model to reduce the over-fitting. There are a range of variable selection techniques that can be used, but the method used in this work is the lasso^9^ (least absolute shrinkage and selection operator). The lasso (Eq. 7) introduces an extra term to the regression equation that is the sum of the absolute value of the coefficients multiplied by a tuning parameter (*λ*). This extra term is the lasso penalty, which has the effect of reducing the coefficients towards zero. This effect will be greater the larger the value of the tuning parameter. If the tuning parameter is equal to zero, the equation reverts to the standard equation for multiple linear regression.

7 $$\sum _{i=1}^{n}\,{({y}_{i}-{\beta }_{0}-\sum _{j=1}^{p}{\beta }_{j}{x}_{ij})}^{2}+\lambda \sum _{j=1}^{p}\,|{\beta }_{j}|=RSS+\lambda \sum _{j=1}^{p}\,|{\beta }_{j}|$$

#### Statistical models

To analyze whether the resonant frequency data could be used to predict the tensile properties of the samples, multiple linear regressions were fitted to the frequencies of the resonant peaks (predictors) and each tensile property (response variable). The predictors were taken to be either the set of measured resonant frequencies for as built condition or the annealed condition. Given that the training set size was only 15 and there are 8 predictors (resonant frequencies) for the as built condition, and 17 predictors for the annealed condition, variable selection was performed using the lasso.

In this analysis, the linear model with the lasso is calculated by co-ordinate descent using the Glmnet library^10^ in the R programming language. The near-optimum tuning parameter is determined by calculating the lasso for a range of discrete values, and using LOOCV to calculate which value of *λ* gives the lowest estimate of test MSE. 1000 different values of *λ* between 10^3^ and 10^−3^ were tested in descending order, as given by (8).

8 $$\lambda ={10}^{(3-\frac{6n}{999})},\,\,n=0,1,2,\ldots ,999$$

The ordinary least squares (OLS) multiple linear regressions and the multiple linear regressions with the lasso were calculated using the as built predictors. As the number of annealed predictors is greater than the training set size, only the multiple linear regressions with the lasso were calculated using these predictors.

## Results

### Experimental results

#### Tensile tests

The results of the room temperature tensile testing are presented in Table 3. AMS4999A^11^ specifies minimum values in the Z-direction for additively manufactured Ti-6Al-4V of 855 MPa for ultimate tensile strength (UTS), 765 MPa for yield strength at 0.2% offset, and 5% elongation at failure. It can be seen from Table 3 that all samples met the minimum requirements for UTS and yield stress, but that seven of the samples failed to meet the required standard for elongation.

**Table 3:** The tensile test results.

ID	Young’s (GPa)	0.2% Yield (MPa)	UTS (MPa)	Elongation (%)	RA (%)
FCE B1	122	1008	1164	7.25	10.20
FCE B6	108	993	1153	3.12	5.42
FCE B8	110	996	1128	2.25	2.97
FCE B10	109	988	1112	4.12	4.44
FCE B14	128	1012	1157	6.00	3.94
FCE B30	118	1007	1134	5.44	5.42
FCE B31	122	1010	1165	6.56	14.86
FCE B33	125	1019	1163	5.69	4.91
FCE B35	125	1015	1162	5.75	2.98
FCE B38	124	1014	1157	6.31	3.47
FCE B44	109	1004	1158	3.19	3.96
FCE B47	113	997	1156	2.88	4.44
FCE B51	121	1052	1165	4.56	6.88
FCE B56	115	994	1146	5.44	3.46
FCE B60	115	998	1119	3.50	3.96

#### Resonant frequency measurements

The measured resonant frequency data for the as built state can be found in Table 4 and for the annealed state in Table 5. It should be noted that for one of the parts (FCE B6) one of the resonant frequency peaks could not be detected in the annealed condition. This part was excluded from the training set for the annealed model, which led to different training sets for the two conditions. It is for this reason that two null models were calculated for each tensile property.

**Table 4:** The measured resonant frequencies in kHz for the parts in the as built condition.

ID	AB1	AB2	AB3	AB4	AB5	AB6	AB7	AB8
FCE B1	20.157	24.166	53.214	83.079	91.978	101.291	147.596	222.719
FCE B6	20.208	24.139	52.968	82.828	91.876	101.163	147.380	220.547
FCE B8	20.162	24.101	52.968	82.602	91.641	100.869	147.019	220.392
FCE B10	20.103	24.076	52.805	82.377	91.377	100.344	146.611	220.668
FCE B14	20.174	24.166	53.132	82.933	91.912	101.198	147.478	221.415
FCE B30	20.163	24.117	52.932	82.619	91.608	100.813	147.046	221.351
FCE B31	20.292	24.248	53.314	83.264	92.137	101.515	147.836	222.095
FCE B33	20.259	24.244	53.196	83.145	92.104	101.251	147.810	222.819
FCE B35	20.270	24.243	53.324	83.105	92.111	101.468	147.824	222.457
FCE B38	20.169	24.183	53.187	83.045	92.041	101.223	147.574	222.437
FCE B44	20.256	24.223	53.323	83.158	92.137	101.474	147.824	222.598
FCE B47	20.279	24.218	53.223	83.049	92.071	101.236	147.694	221.391
FCE B51	20.338	24.281	53.351	83.317	92.124	101.646	147.956	222.880
FCE B56	20.257	24.191	53.296	83.062	92.058	101.399	147.704	222.035
FCE B60	20.173	24.187	53.041	82.880	91.839	100.947	147.406	222.155

**Table 5:** The measured resonant frequencies in kHz for the parts in the annealed condition.

ID	HT1	HT2	HT3	HT4	HT5	HT6	HT7	HT8	HT9	HT10	HT11	HT12	HT13	HT14	HT15	HT16	HT17
FCE B1	7.836	20.885	54.672	78.505	85.134	94.277	120.475	151.336	191.879	198.125	206.005	221.023	223.538	229.338	393.794	397.594	521.384
FCE B6	7.960	20.925	54.741	78.658	84.994	94.288	120.391	151.387	191.573	197.641	206.073	220.878		228.873	393.181	396.744	520.452
FCE B8	7.999	20.865	54.632	78.455	84.791	94.066	120.083	151.079	191.349	197.495	205.648	220.586	223.075	228.490	392.721	396.512	519.752
FCE B10	7.941	20.991	54.552	78.310	84.658	93.914	119.933	150.811	191.168	197.445	205.394	220.373	222.782	228.283	392.619	396.356	519.752
FCE B14	7.893	20.873	54.762	78.654	85.097	94.324	120.520	151.432	192.043	198.029	206.126	221.137	223.667	229.233	393.851	397.674	521.264
FCE B30	8.118	20.856	54.637	78.466	84.818	94.054	120.143	151.086	191.738	197.772	205.703	220.759	223.100	228.784	393.280	397.123	520.439
FCE B31	7.929	20.853	54.819	78.667	85.257	94.383	120.628	151.490	192.033	198.086	206.154	221.211	223.722	229.424	394.303	397.941	521.957
FCE B33	7.899	20.853	54.756	78.612	85.158	94.338	120.576	151.425	191.950	198.193	206.073	221.115	223.609	229.293	393.968	397.654	521.483
FCE B35	7.918	20.990	54.958	78.894	85.166	94.405	120.605	151.528	191.840	198.271	206.169	221.052	223.856	229.097	393.692	397.312	521.139
FCE B38	8.136	20.863	54.787	78.664	85.106	94.330	120.487	151.373	192.078	198.145	205.974	220.980	223.656	229.275	393.871	397.614	521.390
FCE B44	7.932	21.022	54.956	78.746	85.184	94.402	120.596	151.493	191.956	198.213	206.131	221.137	223.723	229.210	393.906	397.684	521.470
FCE B47	7.881	20.947	54.837	78.727	85.076	94.324	120.446	151.384	191.749	198.828	205.990	220.889	223.583	229.004	393.355	396.980	520.725
FCE B51	7.879	20.937	54.867	78.698	85.261	94.334	120.669	151.477	191.945	198.304	206.189	221.208	223.697	229.394	394.345	398.122	521.907
FCE B56	7.965	20.890	54.810	78.713	85.097	94.328	120.521	151.444	191.647	198.163	206.088	220.980	223.633	228.921	393.443	397.062	520.835
FCE B60	7.923	20.910	54.790	78.512	84.935	94.126	120.306	151.197	191.991	198.110	205.818	220.873	223.281	229.111	393.641	397.654	521.154

### Statistical model results

**Figure 4:**
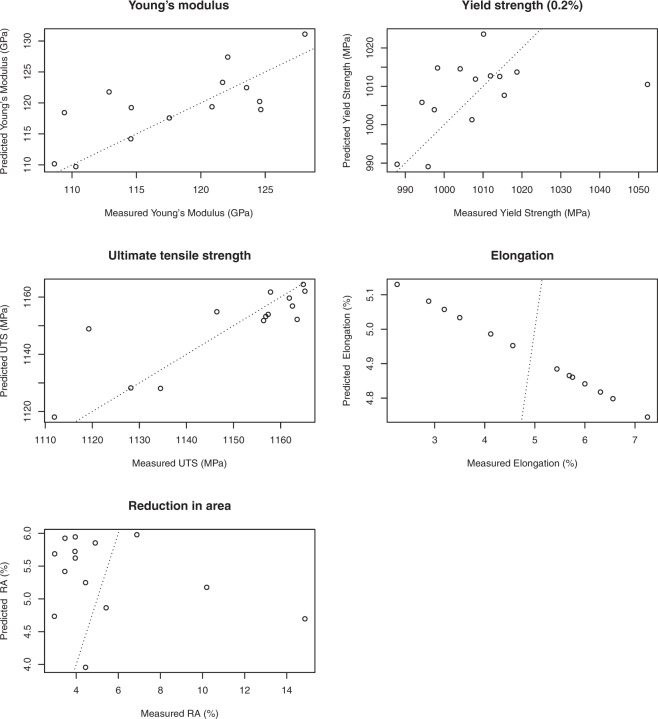
The tensile properties from LOOCV as predicted by using multiple linear regression with the lasso from the resonant frequency peaks in the annealed condition. The dotted lines show where the predicted value is equal to the actual value.

The number of coefficients (predictors) used in each of the fifteen models, along with the estimated test MSE, and the predicted *R*^2^ are presented in Tables 6–10. Tables 6–10 also include the calculated value of *R*^2^ of each model for comparison to the other model statistics. The prediction accuracies are presented graphically in Fig. 4, which for each tensile property plots the value of the response variable as predicted by the model built with that sample excluded against the actual value of the response variable. A line is also included in the plot to indicate where the points should lie if the predicted value equals the actual value.

**Table 6:** Young’s modulus predictive model performance. AB: as built predictors. Ann: annealed predictors. OLS: ordinary least squares.

	AB Null	AB OLS	AB Lasso	Ann Null	Ann Lasso
Coeffs	0	8	2	0	12
*MSE*	48.2	99.4	38.1	43.8	20.0
${R}_{PRED}^{2}$	—	−1.06	0.21	—	0.54
*R* ^2^	—	0.56	0.33	—	0.99

**Table 7:** Yield strength predictive model performance. AB: as built predictors. Ann: annealed predictors. OLS: ordinary least squares.

	AB Null	AB OLS	AB Lasso	Ann Null	Ann Lasso
Coeffs	0	8	3	0	2
*MSE*	257.6	397.8	195.7	260.9	190.9
${R}_{PRED}^{2}$	—	−0.54	0.24	—	0.27
*R* ^2^	—	0.79	0.49	—	0.52

**Table 8:** UTS predictive model performance. AB: as built predictors. Ann: annealed predictors. OLS: ordinary least squares.

	AB Null	AB OLS	AB Lasso	Ann Null	Ann Lasso
Coeffs	0	8	2	0	2
*MSE*	325.6	267.8	95.0	351.1	90.3
${R}_{PRED}^{2}$	—	0.18	0.71	—	0.74
*R* ^2^	—	0.82	0.78	—	0.85

**Table 9:** Elongation predictive model performance. AB: as built predictors. Ann: annealed predictors. OLS: ordinary least squares.

	AB Null	AB OLS	AB Lasso	Ann Null	Ann Lasso
Coeffs	0	8	2	0	0
*MSE*	2.5	5.1	2.2	2.5	2.5
${R}_{PRED}^{2}$	—	−1.00	0.14	—	0.00
*R* ^2^	—	0.51	0.34	—	0.00

**Table 10:** Reduction in area predictive model performance. AB: as built predictors. Ann: annealed predictors. OLS: ordinary least squares.

	AB Null	AB OLS	AB Lasso	Ann Null	Ann Lasso
Coeffs	0	8	0	0	1
*MSE*	10.9	28.8	10.9	11.8	11.5
${R}_{PRED}^{2}$	—	−1.65	0.00	—	0.02
*R* ^2^	—	0.69	0.00	—	0.12

## Discussion

The results for all six models indicate that a multiple linear regression using resonant frequency peaks cannot be used to make predictions about elongation at failure or reduction in area. The two ordinary least squares models have predicted *R*^2^ values less than zero. Two of the models using the lasso have reduced to the null model with no non-zero coefficients, and the remaining two models that use the lasso have predicted *R*^2^ values of just 0.14 and 0.02.

For the Young’s modulus, the yield strength, and the ultimate tensile strength, the models have varying levels of predictive capability. The ordinary least squares models using the as built predictors for the Young’s modulus and the yield strength performed worse than the null model, having predicted *R*^2^ values of −1.06 and −0.54. The equivalent model for predicting ultimate tensile strength performed better, having a predicted *R*^2^ value of 0.18. For the models built using the as built predictors with the lasso, the predictive accuarcy was increased for all three tensile properties, with the model for ultimate tensile strength again demonstrating the greatest predictive accuracy. For the Young’s modulus, the yield strength, and the ultimate tensile strength, the multiple linear regressions that gave the lowest prediction errors were those using the annealed frequency peaks with the lasso. Of these three tensile properties, the Yield strength model predictions had the greatest error, with only a modest improvement over the null model (${R}_{PRED}^{2}=0.27$). The model using the annealed predictors showed some ability in being able to predict the Young’s modulus with ${R}_{PRED}^{2}=0.54$. This would suggest, given the better performance of the UTS model, that although resonant frequency peaks can be used to make predictions about the Young’s modulus of additively manufactured Ti-6Al-4V, a multiple linear regression using resonant frequency peaks is insufficient to properly model the relationship (bias error).

The models for predicting the ultimate tensile strength had the lowest error of all the multiple linear regressions. The model using the annealed predictors achieved ${R}_{PRED}^{2}=0.74$, suggesting that this model may be useful for predicting the UTS. It is unclear whether the variance unexplained by the model is due to the inherent distribution of the response variable (variance error), or whether there is an element of bias error, either due to an incorrect model type, or the exclusion of predictors other than resonant frequency — for example the part temperature or mass.

It should be noted that for all of the models that have at least one non-zero coefficient that the value of *R*^2^ is greater than the value of predicted *R*^2^. For some of the models the values of the two measures of *R*^2^ are sufficiently close that the same interpretation of predictive ability would be reached from either measure. For example, the model to predict UTS using the as built predictors with the lasso has an *R*^2^ of 0.78 and a predicted *R*^2^ value of 0.71. It is quite possible that both of these results could be read to suggest that the model has some predictive capability. Similarly, the model to predict the reduction in area using the annealed predictors with the lasso has an *R*^2^ value of 0.12 and a predicted *R*^2^ value of 0.02. Again it is reasonable to assume that both of these results would be interpreted in the same way: this model has no predictive ability. However, for the ordinary least squares model using the as built predictors to predict reduction in area, the *R*^2^ value of 0.69 could be understood to say that the model does have some predictive use, whereas the predicted *R*^2^ value of −1.65 clearly indicates that no predictive use should be made of the model. This final example illustrates why the authors believe that predicted *R*^2^ should be used in place of *R*^2^ when considering the ability of a statistical model to make predictions.

## Conclusion

It has been demonstrated that *R*^2^ should not be used to measure the ability of multiple linear regressions to make predictions, as low residual error between model and training data does not necessarily translate to low prediction error when used with test data. The predicted *R*^2^ value has been introduced, which is calculated using leave-one-out cross-validation, and is a more useful measure of the prediction accuracy of a statistical model. The calculated value of predicted *R*^2^ has been used to assess the predictive ability of a series of linear models using resonant frequency peaks to predict the mechanical properties of samples built using selective laser melting.

The statistical results indicate that the resonant frequency peaks as measured in the samples either in the as built or the annealed states can be used to make predictions about the ultimate tensile strength of the samples. It should be recognized that although these two linear models were able to explain the majority of the variability in the measured values of UTS, the residual errors are not insignificant when compared to the limited scatter in the measured UTS.

The linear models were less able to accurately predict the measured value of Young’s modulus than the UTS. However, a predicted *R*^2^ value of 0.54 using the annealed resonant frequency data does suggest the possibility that a different type of model could potentially make useful predictions about Young’s modulus using resonant frequency measurements.

There is very limited evidence to support the conclusion that resonant frequency measurements can be used with a linear model to predict yield strength. There is no evidence that a linear model can use resonant frequency data to predict either elongation at failure or reduction in area. It is slightly disappointing that the models were unable to make useful predictions about elongation or reduction in area, as these were the properties that were found to have the most variability in the mechanical testing results.
